# *In Vivo* Mapping of Catecholaminergic Loss and Iron Deposition in Huntington's Disease

**DOI:** 10.1002/mds.70221

**Published:** 2026-02-12

**Authors:** Edoardo R. de Natale, Heather Wilson, Efstratios Karavasilis, Athanasia Liozidou, Chrisoula Kartanou, Georgios Velonakis, Jens Kuhle, Ioannis Zalonis, Georgia Karadima, Leonidas Stefanis, Georgios Koutsis, Marios Politis

**Affiliations:** ^1^ Neurodegeneration Imaging Group University of Exeter Medical School London UK; ^2^ Research Unit of Radiology and Medical Imaging, 2nd Department of Radiology Attikon General University Hospital, School of Medicine, National and Kapodistrian University of Athens Athens Greece; ^3^ 1st Department of Neurology, School of Medicine National and Kapodistrian University of Athens, Aiginition Hospital Athens Greece; ^4^ Research Center for Clinical Neuroimmunology and Neuroscience Basel (RC2NB) University Hospital Basel, University of Basel Basel Switzerland

**Keywords:** Huntington's disease, magnetic resonance imaging, neurodegeneration, neuromelanin, quantitative susceptibility mapping

## Abstract

**Background:**

The pathophysiology of Huntington's disease (HD) remains obscure. Magnetic resonance imaging (MRI) can reveal in vivo molecular changes related to disease pathology.

**Objectives:**

To investigate catecholaminergic neuronal integrity and subcortical brain iron accumulation in HD employing neuromelanin‐sensitive MRI, and quantitative susceptibility mapping (QSM‐MRI).

**Methods:**

Twenty‐five HD gene expansion carriers (HDGECs; 11 premanifest, mean predicted phenoconversion time 21.25 ± 8.28 years) and 25 healthy controls (HC) underwent clinical investigation, measurement of plasma neurofilament light chain (NfL) levels, neuromelanin‐sensitive, and QSM‐MRI on a 3 T scanner. For neuromelanin analysis, the substantia nigra (SN) and locus coeruleus (LC) areas and contrast‐to‐noise ratio (CNR) were computed. Regions of interest from the MuSus100 atlas were segmented for QSM‐MRI analysis, and susceptibility values extracted using the whole brain mask as reference.

**Results:**

Manifest HDGECs showed, on neuromelanin‐sensitive MRI analysis, reduced LC and SN areas, and LC CNR compared with HC (*P* < 0.001). QSM analysis demonstrated increased susceptibility values in caudate, putamen, external and internal pallidum, and the subthalamic nucleus of manifest HGDECs (*P* < 0.001). Higher susceptibility values in these regions correlated with clinical markers of disease burden, higher plasma NfL, and poorer neuropsychological outcomes in multiple domains (*P* < 0.05). Smaller LC area correlated with higher plasma NfL (*P* = 0.029). Higher susceptibility values in the caudate and putamen correlated with lower SN CNR (*P* < 0.05).

**Conclusions:**

Our findings confirm widespread iron subcortical accumulation in HD, reveal significant noradrenergic neuronal loss, and describe dopaminergic alteration which may reflect ongoing pathology along the nigrostriatal pathway. © 2026 The Author(s). *Movement Disorders* published by Wiley Periodicals LLC on behalf of International Parkinson and Movement Disorder Society.

## Introduction

There is a pressing need for in vivo biomarkers able to recapitulate the biological history of Huntington's disease (HD). Magnetic resonance imaging (MRI) techniques are particularly suited as they can non‐invasively probe microstructural and molecular brain changes with high precision,^1^ thus offering promising avenues for in vivo biomarker discovery.

Several studies suggest that the substantia nigra pars compacta (SNc) may be altered in HD,^2^, ^3^, ^4^ although there is disagreement regarding the extent of dopaminergic cell loss.^4^, ^5^ This may functionally reflect impaired nigrostriatal dopamine transmission. Striatal neurons from HD gene expansion carriers (HDGECs) show decreased immunostaining for tyrosine hydroxylase, the rate‐limiting enzyme in dopamine synthesis.^6^ Additionally, molecular imaging studies for the presynaptic dopaminergic system reported loss of striatal dopaminergic terminals and of synaptic vesicles in manifest HDGECs, although without correlation to disease severity.^7^, ^8^, ^9^, ^10^ At a clinical level, reduction of dopaminergic neurotransmission has been associated with juvenile‐onset HD,^11^ in which an akinetic‐rigid syndrome constitutes a predominant feature.^12^ Conversely, fewer and contrasting HD reports exist on the integrity of the locus coeruleus (LC), the brain's primary noradrenergic nucleus.^13^, ^14^

Neuromelanin is a byproduct of catecholaminergic metabolism and an endogenous marker of SNc and LC neuronal density.^15^ When bound to iron, neuromelanin displays paramagnetic properties and can be visualized in vivo using specialized T1‐weighted fast spin‐echo MRI.^16^ A recent study employed this sequence in a small cohort of manifest HDGECs detecting reductions in SN area and neuromelanin signal compared with controls.^17^ However, these findings have not yet been validated in premanifest HDGECs or in larger, clinically well‐characterized cohorts.

Iron dysregulation also plays a central role in HD pathophysiology.^18^ Both in vitro and preclinical studies indicate mutant Huntingtin (mHtt) as being critical in disrupting iron homeostasis,^19^ predisposing HD brains to ferroptosis, an iron‐dependent form of regulated cell death. Excess iron also represents a strong pro‐inflammatory trigger. A post mortem investigation on HD brain tissues revealed that increased iron staining co‐localized with areas of increased reactive astrocytes.^20^

Quantitative susceptibility mapping (QSM) quantitatively estimates tissue magnetic susceptibility.^21^ Susceptibility values show a strong correlation with post mortem histological measures of grey matter iron concentration,^22^ offering a reliable, non‐invasive method for regional iron quantification. Previous studies using this sequence in HD reported elevated susceptibility values, indicative of iron overload, in the putamen, pallidum, and caudate nuclei in both manifest and premanifest HDGECs.^23^, ^24^, ^25^

We employed a multimodal MRI approach combining neuromelanin‐sensitive MRI and QSM to assess catecholaminergic neuronal loss, and iron deposition over an extensive range of subcortical regions of interest (ROIs) in premanifest and manifest HDGECs. To evaluate the pathophysiological underpinning and the translational relevance of these imaging markers, we examined the correlation between QSM and neuromelanin markers, and their relationship to established clinical indicators of disease burden, motor, neuropsychiatric, and cognitive performance, and plasma levels of neurofilament light chain (NfL), a fluid biomarker of neurodegeneration.

## Methods

### Study Participants

This was a cross‐sectional study performed at the 1st Department of Neurology, Aiginition Hospital of the National and Kapodistrian University of Athens, Greece. A total of 25 HDGECs with genetic confirmation of a pathological cytosine‐adenine‐guanine (CAG) expansion (>40 repeats) in exon 1 of the *HTT* gene participated in this study.

Fourteen participants with a Unified Huntington's Disease Rating Scale‐Total Motor Score (UHDRS‐TMS) ≥ 7, a Total Functional Capacity (UHDRS‐TFC) ≤ 12, and a Diagnostic Confidence Scale (DCL) of 4 were labeled as manifest HDGECs. Eleven participants with UHDRS‐TMS ≤6, a TFC of 13, DCL of 0, and CAG‐Age Product (CAP) scores either above or below 70 were classified as premanifest HDGECs. In premanifest HDGECs, predicted years to phenoconversion were estimated using the Langbehn formula.^26^ Twenty‐five age‐ and sex‐matched participants with no personal or family history of neurological disorders participated as HC.

Exclusion criteria included known, clinically significant, systemic comorbidities, history of neurological conditions such as stroke, hemorrhage or demyelinating conditions, pregnancy, and general contraindications to MRI scanning.

The study was approved by the Research Ethics Committee of Aiginition Hospital and the Hospital Board (Ethics ID: 445/18‐06‐2018) and all participants gave their written informed consent before enrollment. All study procedures were performed according to the Declaration of Helsinki and Good Clinical Practice.

### Clinical Assessments

At screening, all participants underwent collection of demographic information including age, education, symptom onset, and family medical and psychosocial history. In HDGECs, disease burden was assessed using the Disease Burden Score (DBS) and the CAP score, computed as: age × (CAG–30)/6.49.^27^ On the same day, participants completed a battery of motor, neuropsychiatric, and neuropsychological tests assessing language, working memory, attention, information and processing speed, executive function, verbal and visuospatial memory learning, and identity and emotion matching. A comprehensive list of the clinical and neuropsychological tests administered is reported in the Supporting Information.

### MRI Acquisition

All participants underwent one 32‐channel head coil 3 T MRI scan on a Philips Achieva Scanner (Philips Healthcare, Best, The Netherlands) for acquisition of the neuromelanin‐MRI and QSM sequences, in the same session. The neuromelanin‐MRI parameters were as follows: TR/TE: 829/17.3 ms; slices: 12; slice thickness: 2.5 mm; voxel size: 0.8 × 0.8 mm; FoV: 320 × 182 × 41 mm^3^; flip angle: 123 ; acquisition time: 5:20 min. A three‐dimensional (3D) multi‐echo gradient‐recalled echo (GRE) sequence was used for QSM with the following parameters: TR/TE1: 3.8/4.5 ms; ΔTE: 4.4 ms, 8 echoes, flip angle: 20°, voxel size: 1 × 1 × 1 mm^3^, FOV: 240 × 240 × 150 mm^3^, pixel bandwidth: 333 Hz, SENSE factor: 2 × 1, transverse slab, total acquisition time: 9:04 min. A standardized T1‐weighted 3D turbo field echo sequence was acquired for anatomical referencing and automated image segmentation, using the following parameters: TR/TE: 9.9/3.8 ms, flip angle: 7°, voxel size: 1 × 1 × 1 mm^3^, acquisition time: 5:39 min. Both magnitude and phase images were saved for QSM reconstruction, and images were preliminarily examined for any presence of image artifacts or abnormalities.

### Blood Collection

All participants donated a sample of venous blood in non‐fasting mode, in a 4 mL EDTA tube for quantification of plasma levels of NfL. The whole‐blood samples were centrifuged within 2 hours of collection and then aliquoted into 0.5 mL Sarsted tubes for storage in −80°C freezers. The quantification of NfL was performed at the University of Basel, Switzerland. Plasma samples were thawed and analyzed for NfL concentrations using the single molecule array (SIMOA) Neurology 2‐Plex B assay (Quanterix, Billerica, MA, USA) on the HDx platform, in accordance with the manufacturer's instructions, between x and y.^28^ All NfL values were within the linear ranges of the assays.

### Neuromelanin‐Sensitive MRI Analysis Methods

To calculate the SN and LC contrast‐to‐noise ratio (CNR) and areas, two different sets of ROIs were delineated on each subject native space using Analyze Software v.14.0 (Analyze, Biomedical Imaging Resources, Mayo Clinic, Rochester, MN, USA) by a neurologist with 8 years of experience in neuroimaging analysis. To calculate the SN intensity and CNR, we manually placed two symmetric 2.4 mm squares bilaterally on the medial and the lateral SN and two 4.0 mm squares bilaterally over the cerebellar peduncles as SN reference regions. To calculate the LC intensity and CNR, we placed two bilateral and symmetric 2.4 mm squares on the hyperintensity area of the posterior pons adjacent to the lateral floor of the fourth ventricle, and a 5.6 mm reference region over the middle of the brainstem (Fig. S1). This procedure was performed twice on each subject.

To calculate the SN and LC areas, we manually delineated the SN and LC hyperintense regions on the same slices to sample the areas using Analyze v.14.0 (Fig. S2). This procedure was also performed twice.

We performed a two‐way intraclass correlation coefficient to assess the reliability of the two SN and LC CNR and area measurements, which showed alpha values ranging from 0.782 to 0.917, an expression of a good‐to‐excellent reliability (Table S1). We then averaged the CNR values obtained from the two measurements to obtain a final CNR, which was used for analysis. The SN and LC area ROIs were then merged using FMRIB Software Library (FLS) fslmaths utility to obtain final ROIs, which were sampled and left‐ and right‐averaged to obtain a single value for analysis.

### QSM‐MRI Analysis

QSM images were processed using the SEPIA toolbox (v.1.2.2.6)^29^ in Matlab v2018b, using the Laplacian‐based phase unwrapping method within the MEDI toolbox. The V‐SHARP method for background field removal and the QSM algorithm iterative LSQR was used to create a magnetic susceptibility Chimap, in parts per million applying the brain mask, and the cerebrospinal fluid (CSF) mask, as the reference tissue.

For quantification of regional susceptibility, subcortical structural segmentation was performed. The multimodal‐fused magnetic susceptibility (MuSus‐100) atlas was used to define a list of ROIs as: caudate, putamen, external, internal and ventral globus pallidus, substantia nigra pars compacta and pars reticulata, subthalamic nucleus, and red nucleus. Manual corrections on the caudate were applied in Analyze v.14.0 to improve atlas fitting around the caudate, avoiding sampling into the ventricle.

All susceptibility values are reported relative to the mean susceptibility value of the reference region. The brain mask was selected as the primary reference tissue with CSF mask as reference tissue (results reported in Table S5).

Full analysis pipelines for both MRI sequences are described in the Supporting Information.

### Statistical Methods

A preliminary Shapiro–Wilk test was performed to assess normality. Group differences in demographic and clinical characteristics were analyzed using the Student's *t*‐test (for parametric variables) and Mann–Whitney U test (for nonparametric variables) as appropriate. Categorical variables were analyzed with the *χ* ^2^ test. Between‐group differences in neuromelanin and QSM‐MRI data across the three groups of manifest HDGECs, premanifest HDGECs, and HC were assessed using multivariate analysis of covariance (MANCOVA) with age and sex as covariates and post‐hoc Bonferroni correction. The Bonferroni threshold was set as *P* = 0.0125 for the neuromelanin‐sensitive MRI parameters and *P* = 0.005 for the QSM‐MRI parameters. Correlations across imaging modalities and between imaging parameters and clinical characteristics in the HDGECs cohort were performed using Pearson's partial correlation, controlling for age and sex and applying the Benjamini–Hochberg false discovery rate (FDR) correction with q‐values of <0.05. To correlate imaging data with performances on neuropsychological tests, education was added as the controlling factor. Since CAP score is a function of age, we used Pearson's linear correlation to compute correlations between this measure and imaging variables. Correlations with UHDRS‐TMS, UHDRS‐TFC, Physical Performance Test (PPT), and disease duration were performed in the manifest HDGEC subgroup only, due to score invariance in the premanifest group.

Statistical analysis and graph illustration were performed using Statistical Package for Social Science version 28.0 (SPSS Inc., Chicago, IL, USA), and GraphPad Prism (version 10.0). Significativity was set as *P* < 0.05. All values are reported as mean ± standard deviation and *P‐*values as corrected.

## Results

### Cohort Overview and Clinical Characteristics

A total of 25 HDGECs and 25 HC were included. After quality control and exclusions, the final analysis included: (a) neuromelanin‐sensitive MRI: 23 HC, 10 premanifest HDGECs, 14 manifest HDGECs; (b) QSM‐MRI: 25 HC, 10 premanifest HDGECs, 13 manifest HDGECs; and (c) NfL analysis: 25 HC, 10 premanifest HDGECs, 12 manifest HDGECs.

Demographic and clinical characteristics are summarized in Table 1. Both premanifest and manifest HDGECs had fewer years of education compared with HC (*P* < 0.001). Manifest HDGECs performed worse than both HC and premanifest HDGECs across all motor, behavioral, and cognitive assessments (all *P* < 0.05), except for the Beck Depression Inventory‐II scale (BDI‐II). Premanifest HDGECs showed higher Huntington's Disease Quality of Life (HDQoL) and Problem Behavior Assessment (PBA) scores, and elevated plasma NfL levels compared with HC (*P* < 0.001). They also showed reduced performances on multiple cognitive tests (Table S2).

**TABLE 1:** Main demographic and clinical characteristics of the three populations

Parameter	HC (n = 25)	Premanifest HDGECs (n = 11)	Manifest HDGECs (n = 14)	*P*‐value HC vs. premanifest HDGECs	*P*‐value HC vs. manifest HDGECs	*P*‐value premanifest HDGECs vs. manifest HDGECs
Age (years)	40.32 ± 11.57	39.36 ± 7.46	46.21 ± 11.26	0.761	0.132	0.076
Gender (M:F)	13:12	4:7	4:10	0.481	0.193	1.00
Education (years)	16.32 ± 2.75	12.72 ± 1.62	11.00 ± 3.55	<0.001	<0.001	0.150
CAG long allele	–	43.18 ± 2.04	44.86 ± 2.85	–	–	0.101
p90 conversion time (years)	–	21.25 ± 8.28	–	–	–	–
Time from diagnosis (years)	–	–	4.43 ± 1.74	–	–	–
DBS	–	297.59 ± 77.93	409.39 ± 71.64	–	–	0.001
CAP score	–	79.21 ± 15.57	102.25 ± 13.96	–	–	<0.001
UHDRS‐TMS	0.00 ± 0.00	1.00 ± 1.73	34.29 ± 18.88	0.207	<0.001	<0.001
UHDRS‐TFC	13.00 ± 0.00	13.00 ± 0.00	9.43 ± 4.11	1.00	<0.001	0.002
UHDRS‐FAS	25.00 ± 0.00	25.00 ± 0.00	17.00 ± 8.29	1.00	<0.001	0.004
UHDRS‐IS	100.00 ± 0.00	100.00 ± 0.00	77.86 ± 20.07	1.00	<0.001	0.001
PPT	36.00 ± 0.00	36.00 ± 0.00	20.29 ± 11.68	1.00	<0.001	<0.001
Finger tapping right	54.04 ± 7.78	51.55 ± 6.74	24.64 ± 8.87	0.341	<0.001	<0.001
Finger tapping left	49.20 ± 8.27	42.90 ± 8.48	19.14 ± 7.68	0.053	<0.001	<0.001
PBA	1.00 ± 0.00	10.64 ± 3.72	52.71 ± 27.40	<0.001	<0.001	<0.001
BDI‐II	3.20 ± 4.19	6.73 ± 6.25	16.14 ± 15.18	0.093	<0.001	0.06
HDQoL	0.00 ± 0.00	41.73 ± 9.49	137.43 ± 51.21	<0.001	<0.001	<0.001
Plasma NfL (pg/ml)	3.16 ± 1.27	11.00 ± 10.28	33.01 ± 11.84	<0.001	<0.001	<0.001

*Note*: Student's *t*‐test for parametric variables, or Mann–Whitney U test for nonparametric variables, and *χ* ^2^ test.

Abbreviations: HC, healthy controls; HDGECs, Huntington's Disease gene expansion carriers; M, male; F, female; CAG, cytosine‐adenine‐guanine; DBS, Disease Burden Score; CAP, CAG‐Age Product; UHDRS, Unified Huntington's Disease Rating Scale; TMS, Total Motor Score; TFC, Total Functional Capacity; FAS, Functional Assessment Scale; IS, Independence Scale; PPT, Physical Performance Test; PBA, Problem Behavior Assessment; BDI‐II, Beck Depression Inventory‐II; HDQoL, Huntington's Disease Quality of Life; NfL, neurofilament light.

### Neuromelanin‐Sensitive MRI Findings

**FIG. 1:**
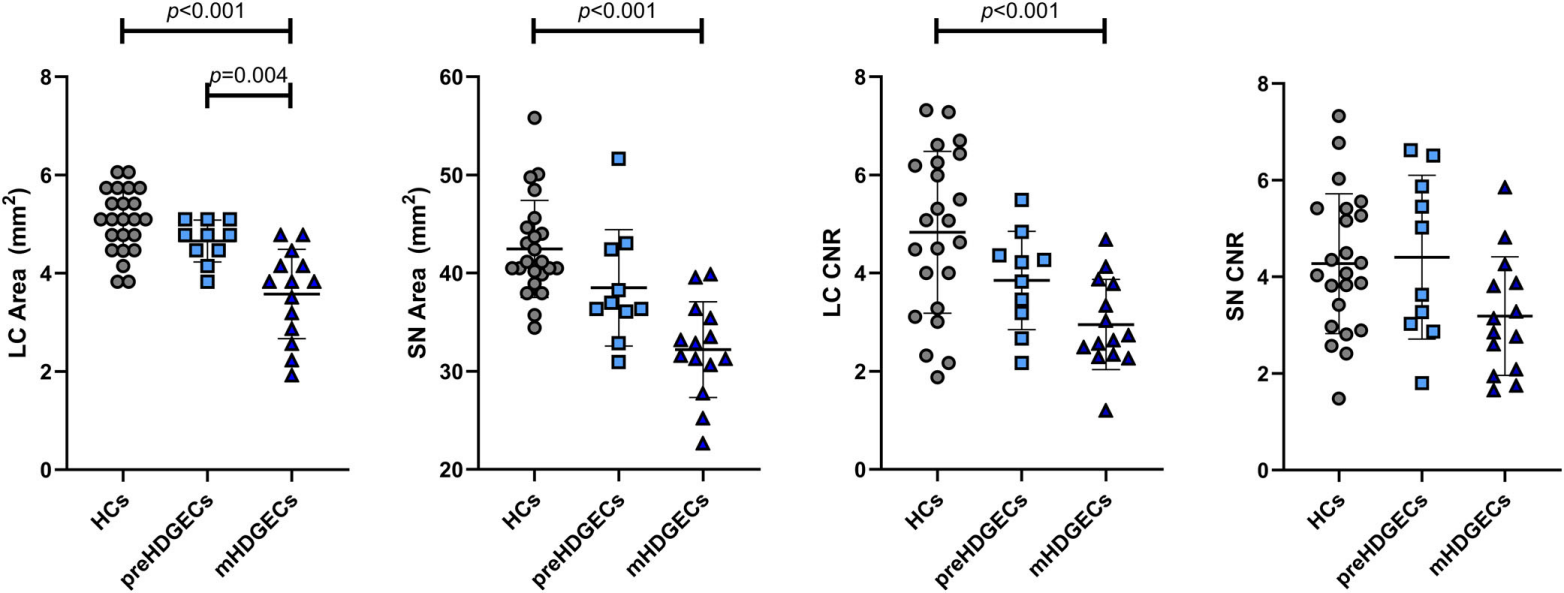
Comparison of the neuromelanin magnetic resonance imaging variables across the three populations studied. Multivariate analysis of covariance, with age and sex as covariates, and with Bonferroni correction. LC, locus coeruleus; HCs, healthy controls; preHD, premanifest Huntington's disease gene expansion carrier; mHD, manifest Huntington's disease gene expansion carrier; SN, substantia nigra; CNR, contrast‐to‐noise ratio. [Color figure can be viewed at wileyonlinelibrary.com]

MANCOVA, using age and sex as covariates and Bonferroni correction, showed significant group effects for the SN area (F_2,42_ = 15.785, *P* < 0.001), the LC area (F_2,42_ = 17.615, *P* < 0.001), and the LC CNR (F_2,42_ = 11.200, *P* < 0.001) (Table S3). Post‐hoc between‐group analysis (Fig. 1) demonstrated reduced SN and LC areas, and LC CNR in manifest HDGECs compared with HC (all: *P* < 0.001). Manifest HDGECs had lower LC area compared with premanifest HDGECs (*P* = 0.004).

### QSM‐MRI Findings

MANCOVA, using age and sex as covariates and after Bonferroni correction, showed significant group effects on susceptibility values in the caudate (F_2,43_ = 25.956, *P* < 0.001), putamen (F_2,43_ = 36.888, *P* < 0.001), internal pallidum (F_2,43_ = 27.483, *P* < 0.001), external pallidum (F_2,43_ = 53.857, *P* < 0.001), and SN (F_2,43_ = 8.693, *P* < 0.001) (Table S4).

**FIG. 2:**
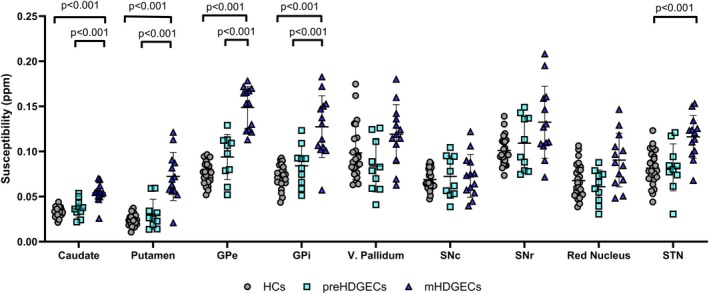
Comparison of the quantitative susceptibility mapping magnetic resonance imaging (QSM‐MRI) susceptibility values across the three populations studied. Multivariate analysis of covariance, with age and sex as covariates, and with Bonferroni correction. GPe, external globus pallidus; GPi, internal globus pallidus; V, ventral; SNc, substantia nigra pars compacta; SNr, substantia nigra pars reticulata; HCs, healthy controls; preHD, premanifest Huntington's disease gene expansion carrier; mHD, manifest Huntington's disease gene expansion carrier. [Color figure can be viewed at wileyonlinelibrary.com]

Post‐hoc between‐group analysis showed increased susceptibility values in manifest HDGECs compared with HC in the SN, external and internal pallidum, caudate, and putamen (*P* < 0.001; Fig. 2). Changes in the red nucleus, SNr, and accumbens did not survive Bonferroni correction.

Premanifest HDGECs exhibited a trend for increased susceptibility values compared with HC in the external pallidum (*P* = 0.059). Manifest HDGECs showed increased susceptibility values compared with premanifest HDGECs in the external, internal and ventral pallidum, caudate, and putamen (*P* < 0.05), with changes in the ventral pallidum that did not survive Bonferroni correction.

Between‐group comparison of QSM‐MRI data using the CSF mask as the reference tissue showed similar results (Table S5).

### Correlations Between QSM‐MRI in the Striatum and Neuromelanin‐Sensitive MRI in the SN

**FIG. 3:**
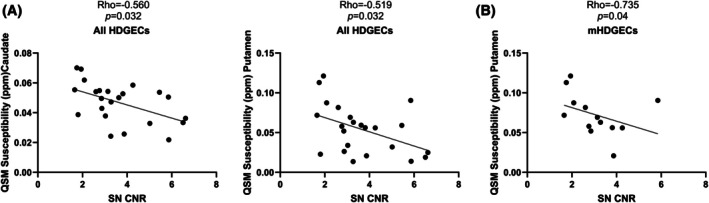
Significant correlations between iron concentration in regions of interest measured by quantitative susceptibility mapping magnetic resonance imaging (QSM‐MRI) and neuromelanin‐MRI parameters in the substantia nigra (SN). (A) Correlations in the whole Huntington's disease gene expansion carriers (HDGECs) group between QSM values in the caudate and putamen and the SN contrast‐to‐noise ratio (CNR). (B) Correlations in the manifest HDGECs (mHDGECs) only, between QSM values in the putamen and the SN CNR. Partial correlation analysis, controlling for age and sex, with Benjamini–Hochberg correction for false discovery rate.

Partial correlation analysis controlling for age and sex revealed, in the HDGECs cohort, a correlation between higher QSM susceptibility values in the caudate and putamen, and lower SN CNR (*P* < 0.05; Fig. 3A). When analyzing the two subgroups of HDGECs we found that after FDR correction this correlation was maintained in the manifest HDGECs in the putamen (rho: 0.735, *P* = 0.04) (Fig. 3B). We did not detect any correlations between QSM susceptibility values in the SNc and any neuromelanin‐sensitive MRI parameters in the SN. Full correlations between SN neuromelanin and QSM‐MRI parameters are available in Table S6.

### Correlations Between Neuromelanin MRI and Clinical Measures

**FIG. 4:**
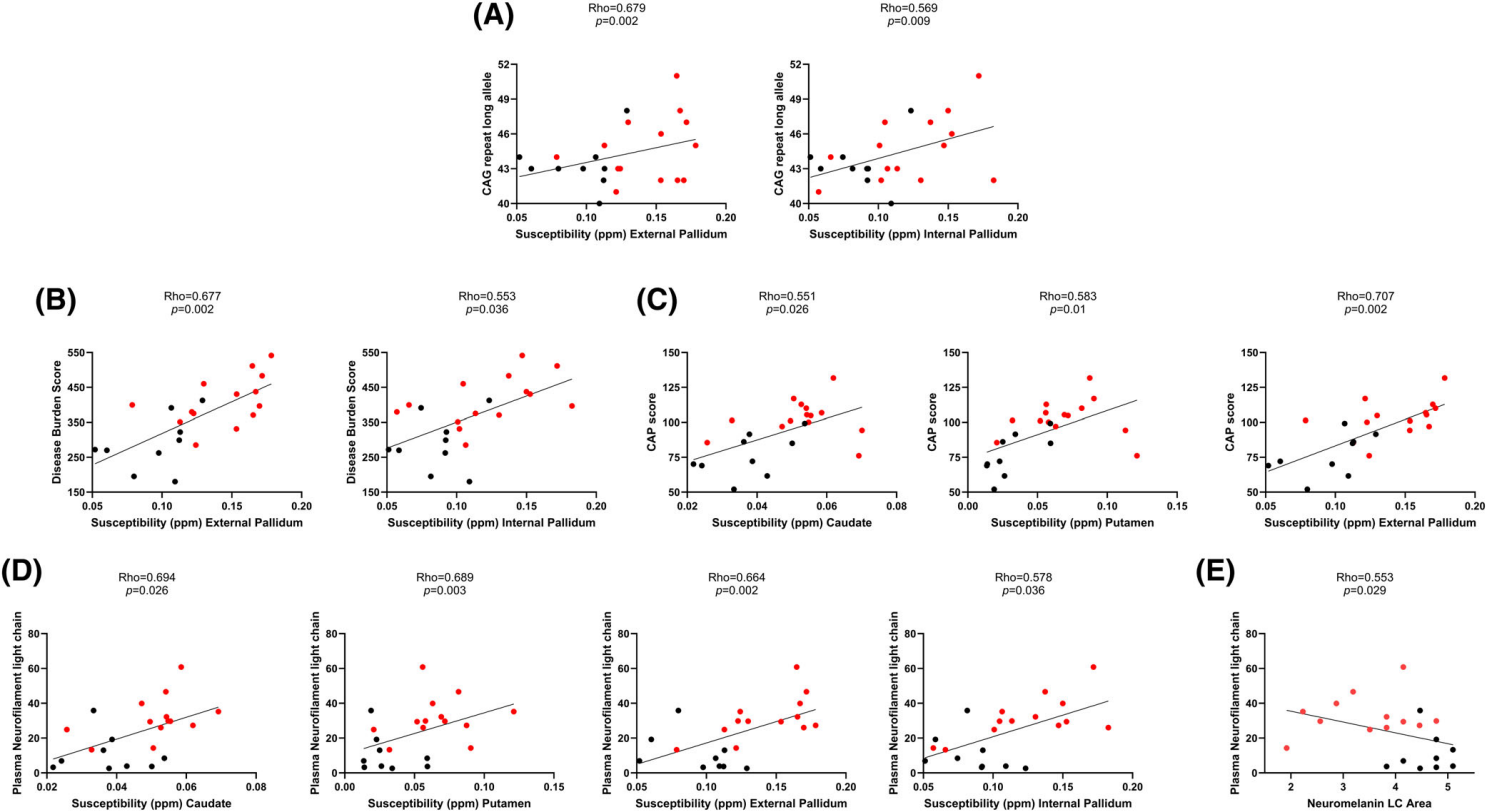
Significant correlations, in all Huntington's disease gene expansion carriers (HDGECs), between neuroimaging parameters and clinical and biological features. (A) Significant correlations between quantitative susceptibility mapping magnetic resonance imaging (QSM‐MRI) susceptibility values in the external and internal pallidum and the length of the cytosine‐adenine‐guanine (CAG) repeat in the long allele. (B) Significant correlations between QSM‐MRI susceptibility values in the external and internal pallidum and increased disease burden score. (C) Significant correlations between QSM‐MRI susceptibility values in caudate, putamen, and external pallidum, and higher CAP score. (D) Significant correlations between QSM‐MRI susceptibility values in caudate, putamen, external and internal pallidum, and higher plasma neurofilament light (NfL) levels. (E) Significant correlations between reduced neuromelanin locus coeruleus (LC) area and higher plasma NfL levels. The red dots indicate manifest HDGECs and the black dots indicate premanifest HDGECs. Partial correlation analysis, controlling for age and sex, with Benjamini–Hochberg correction for false discovery rate. CAP, CAG‐Age Product. [Color figure can be viewed at wileyonlinelibrary.com]

Partial correlation analysis controlling for age and sex and after FDR correction revealed a correlation between smaller LC areas and higher scores on the HDQoL scale (rho: −0.664, *P* = 0.013), the PBA scale (rho: −0.607, *P* = 0.021), lower performances in the finger tapping tests (rho: 0.656, *P* = 0.013 and rho: 0.576, *P* = 0.026), and higher plasma levels of NfL (rho: −0.533, *P* = 0.029; Fig. 4E). An overview of the correlations between neuromelanin‐sensitive MRI and clinical and neuropsychological measures is provided in Tables S7 and S8.

### Correlations Between QSM‐MRI and Clinical Measures

Partial correlation analysis controlling for age and sex and after FDR correction showed a correlation between higher susceptibility values in the external and internal pallidum and larger CAG repeats (*P* < 0.01; Fig. 4A), and higher DBS score (*P* < 0.01; Fig. 4B); higher susceptibility values in the caudate, putamen, and external pallidum also correlated with increased CAP score (*P* < 0.01; Fig. 4C). Furthermore, higher susceptibility values in the caudate, putamen, and external and internal pallidum correlated with higher NfL plasma levels (*P* < 0.01; Fig. 4D). Higher susceptibility values in the caudate, putamen, and external pallidum also correlated with higher scores on the HDQoL scale and with worse performances on the finger tapping test (Table S9).

As regards cognitive tests, higher susceptibility values in the caudate, putamen, and external and internal pallidum correlated with poorer performances in the phonemic and semantic verbal fluency, the oral and written Symbol Digit Modalities Test for divided attention, the Stroop test for complex attention and inhibition of response, and the delayed Hopkins Verbal Learning Test‐Revised for verbal memory and learning (*P* < 0.05). Higher susceptibility values in the external and internal pallidum also correlated with lower Montreal Cognitive Assessment (MoCA) scores, and worse performances in the Digit Span Backwards Test for working memory, the Trial Making Test for cognitive flexibility, the Brief Visuospatial Memory Test‐Revised for visuospatial memory and learning, and the Identity Matching Test for identity and emotion matching (*P* < 0.05; Table S10).

## Discussion

We report, employing neuromelanin‐sensitive and QSM‐MRI, a significant reduction in neuromelanin‐related signal in the SN and LC of a group of HDGECs, and marked iron accumulation in several subcortical nuclei. These are linked to disease severity, motor, cognitive, and neuropsychiatric symptoms, and to fluid biomarkers of neurodegeneration, supporting their potential utility as in vivo HD biomarkers with implications for HD pathophysiology.

### Dopaminergic Pathology of the SN in HD

When examining the SN, we detected a significant loss of neuromelanin‐sensitive area, in the absence of changes in neuromelanin‐containing neurons, and of increased iron content. We did, however, find a correlation between increased iron in the caudate and putamen and a decrease in neuromelanin‐positive SN dopaminergic cells as expressed by the SN CNR. This prompts some considerations on the pathophysiology of the SN and its dopaminergic connections in HD.

There is active debate about the existence and the meaning of nigrostriatal dopaminergic pathology in HD. Previous morphometric MRI studies indicated an atrophy of the SN in HD,^2^, ^8^, ^30^ and molecular imaging studies employing the dopaminergic tracers [^11^C]β‐CIT, [^123^I]FP‐CIT, or [^11^C]DBTZ, revealed a pattern of presynaptic dopaminergic terminal loss^7^, ^8^, ^9^, ^10^ which significantly progressed over time.^31^ By contrast, animal and human studies showed mixed findings on altered SNc dopaminergic cells count,^2^, ^4^, ^5^ and a [^18^F]DOPA positron emission tomography (PET) study on only three patients reported conflicting data on the capacity of dopamine synthesis in HD.^32^

These studies suggest that in HD there may be a structural atrophy of the SN, a functional pathology of the presynaptic dopaminergic terminals but a relative functional preservation of the SNc dopaminergic cell bodies. These may deteriorate at a later stage, when dopaminergic depletion may be associated with an akinetic‐rigid phenotype, a feature of advanced HD. Our findings in a group of manifest HDGECs without remarkable akinetic‐rigid symptomatology, in which SNc area loss is accompanied by a relative preservation of neuromelanin‐sensitive MRI markers of dopaminergic cell bodies integrity in the SN, in the absence of significant iron accumulation, support this view. A mismatch between neuromelanin‐MRI and iron‐MRI findings in the SNc is not unexpected. In Parkinson's disease (PD), a disorder characterized by extensive SNc deterioration, combined neuromelanin‐ and iron‐MRI studies reported a presence^33^ or lack^34^, ^35^, ^36^ of correlation between these SNc alterations, suggesting that neuromelanin loss and iron accumulation may occur independently.

In addition to this, we detected a correlation between pathological iron accumulation in the basal ganglia regions and reduced neuromelanin‐positive cellular density in the SN. The SN connects with the putamen and caudate along the dopaminergic nigrostriatal pathway, and mHtt exerts direct toxic effects on axonal transport by means of both retrograde^37^ and anterograde^38^ alteration mechanisms. In our HDGECs, the putamen and caudate are subject to excessive iron accumulation and may represent regions in which the toxic effect of mHtt is stronger and, notably, areas where functional pathology may be spreading to their functionally connected regions. The correlation we have detected between iron accumulation in these areas and SN CNR reduction, significant especially in the manifest group, may represent the effect of an initial, ongoing spreading of HD‐related pathology to other areas, including the SNc. This hypothesis in HD needs to be confirmed by larger, longitudinal studies, designed to better define the trajectory of SNc alteration and its clinical relevance with regard to the emergence, and progression, of akinetic‐rigid motor symptoms.

### Noradrenergic Pathology of the LC in HD

Our manifest HDGECs showed a loss of LC area and CNR, indicative of an ongoing noradrenergic pathology. Additionally, we detected a correlation between smaller LC area and higher NfL plasma levels. The LC is an underreported site of cellular degeneration in HD, and little is known about the role of noradrenergic pathology in this condition. In the past, only a few post mortem HD studies have assessed changes in noradrenaline levels and LC pathology. While one study applying graphical methods did not show any significant changes in the LC neuronal density,^13^ another work reported LC atrophy and cellular loss associated with late‐stage HD and depression, suggesting that noradrenergic degeneration may emerge as a late phenomenon.^14^

No other in vivo studies, such as PET imaging, have investigated LC changes across HD stages. Future studies, including molecular imaging studies targeting noradrenergic terminals, are needed to confirm these results and evaluate the role of LC pathology in HD.

### Pathophysiology of Iron Accumulation in HD

We report extensive pathological iron accumulation in several subcortical regions of manifest HDGECs, and a trend towards an increase in iron content in the external pallidum of premanifest HDGECs. Previous studies on premanifest HDGECs reported iron overload in the caudate and putamen.^23^, ^24^ However, compared with our cohort, those carriers either showed shorter predicted time from phenoconversion^23^ or a higher mean UHDRS‐TMS score of 7.2, indicative of some degree of motor impairment.^24^ A more recent study on premanifest HDGECs subdivided into far‐ and near‐onset, detected increased iron content in the caudate and putamen in the latter, but not the former, subgroup of carriers.^25^ Overall, our findings are in line with the existing literature and expand on the regional burden of iron accumulation across deep, subcortical areas.

Consistent with previous work, we found that increased iron content in the caudate and putamen of HDGECs correlates with higher DBS^23^ and CAP scores^25^, ^39^, ^40^ and higher NfL levels.^41^ We extend these findings by adding correlations between increased iron content also in pallidal regions with biomarkers of disease burden in multiple regions, clinical measures of motor and behavioral symptoms’ impairment, and with poorer cognitive performances in several tests including those for verbal and visual memory, working memory, attention, and language. Iron accumulation has been associated with progression of cognitive impairment across neurodegenerative conditions characterized by dementia, such as PD dementia^42^ or Alzheimer's disease.^43^ In this latter condition, iron deposition may also be spatially associated with protein accumulation.^44^ This evidence suggest a biological role of iron in determining or catalyzing cognitive deterioration. mHtt predisposes the brain to ferroptosis, a widely recognized pathophysiological mechanism of cellular damage in HD.^19^ Overall, our results indicate that iron accumulation contributes significantly to the determination of disease severity in HD. Longitudinal studies are needed to assess its role in mediating disease milestones such as the transition from the premanifest to the manifest stage. Nevertheless, studying the mechanisms of iron accumulation in HD potentially presents relevant translational implications, as preliminarily demonstrated by HD model studies using the iron chelators deferiprone and ferrostatin‐1.^45^, ^46^

Some limitations need to be acknowledged. The sample size was composed of a relatively low number of premanifest and manifest HDGECs. Moreover, the group of premanifest HDGECs was far temporally from the expected age of onset and did not include any near‐onset carriers, thus limiting the possibility of further extrapolating the trajectory of neuromelanin and QSM‐related biomarkers changes across this condition.

In conclusion, this study provides compelling in vivo evidence for two pathological processes in HD: a vulnerability of neuromelanin‐containing catecholaminergic neurons, and widespread iron accumulation across key subcortical structures. These imaging biomarkers, particularly iron accumulation levels, showed robust associations with genetic burden indices, such as CAP and DBS scores, as well as with plasma NfL levels and clinical measures. These findings strengthen the potential of MRI in the in vivo study of HD, providing complementary information about disease pathophysiology from different perspectives.

## Author Roles

(1) Research Project: A. Conception, B. Design, C. Participant Recruitment, D. Research Procedures, E. Imaging Procedures, F. Neurofilament Light Chain (NfL) Measurements; (2) Statistical Analysis: A. Design, B. Data Analysis and Discussion of Initial Results, C. Review and Critique; (3) Manuscript Preparation: A. Writing of the First Draft, B. Review and Critique; (4) A. Secured Project Funding, B. Project Supervision in Athens, C. Supervision of Entire Project.

E.R.d.N.: 2A, 2B, 2C, 3A, 3B.

H.W.: 2B, 2C, 3B.

E.K.: 1E, 3B.

A.L.: 1C, 1D, 3B.

C.K.: 1C, 1D, 3B.

G.V.: 1E, 3B.

J.K.:1F, 3B.

I.Z.: 3B, 4B.

G.Kar.: 3B, 4B.

L.S.: 3B, 4B.

G.Kou.: 3B, 4B.

M.P.: 1A, 1B, 3B, 4A, 4C.

## Financial Disclosures

This study was funded by a philanthropic donation from City Electrical Factors. M.P. research is supported by The Michael J. Fox Foundation for Parkinson's Research, City Electrical Factors, Hoffmann‐La Roche, and Invicro London. J.K. received speaker fees, research support, travel support, and/or served on advisory boards for the Swiss MS Society, Swiss National Research Foundation (320030_212534/1), University of Basel, Progressive MS Alliance, Alnylam, Argenx, Bayer, Biogen, Bristol Myers Squibb, Celgene, Immunic, Merck, Neurogenesis, Novartis, Octave Bioscience, Quanterix, Roche, Sanofi, and Stata DX. G. Kou. has received research grants from Genesis Pharma and Teva; and in the past 36 months consultation fees, advisory board remuneration, and honoraria from Genesis Pharma, Bristol Myers Squibb, and AstraZeneca. All other authors report no conflicts of interest.

## Supporting information




**Data S1** Supporting information

## Data Availability

Data are available upon reasonable request from the Chief Investigator and after signature of a Data Transfer Agreement.
