# Supplementary material for: In Vivo Mapping of Catecholaminergic Loss and Iron Deposition in Huntington's Disease
Source: Mov Disord. 2026 Feb 12;41(5):1257–67. doi: 10.1002/mds.70221 (PMC13206212; doi:10.1002/mds.70221)
Supplement: Supplementary file 1 — Data S1 Supporting information [file MDS-41-1257-s001.docx]

**Supplementary materials for:**

***In vivo* mapping of catecholaminergic loss and iron deposition in Huntington’s disease.**

**Contents:**

|  | **Page** |
| --- | --- |
| **Supplementary Methods** | **3** |
| **Supplementary Figure 1:** Description of the methods used to calculate the contrast-to-noise ratio in neuromelanin MRI images. Squared areas were placed on the left and right loci coerulei, the left and right substantia nigra and the reference regions in two separate sessions (measure #1 and measure #2). The intensity values were extracted and an intraclass correlation coefficient (ICC) was performed. The values from the two measurements were averaged to obtain a single SN CNR and LC CNR to be used for analysis. | **7** |
| **Supplementary Figure 2:** Description of the method used to sample the SN and LC areas in neuromelanin MRI images. The left and right LC and SN areas were manually drawn on a single slice in two separate sessions (measure #1 and measure #2). Their areas were sampled and an intraclass correlation coefficient (ICC) was performed. The left and right LC and SN areas were then averaged to obtain a single SN area and a single LC area that were used for analysis. | **8** |
| **Supplementary Table 1:** Intraclass Correlation Coefficient between the measures #1 and #2 of Neuromelanin regions of interest. | **9** |
| **Supplementary Table 2.** Differences in neuropsychological tests between the three populations studied. | **10** |
| **Supplementary Table 3:** Neuromelanin MRI results across the three populations studied. | **11** |
| **Supplementary Table 4:** Comparison of QSM MRI results, using the whole brain reference mask, across the three populations studied. | **12** |
| **Supplementary Table 5:** Comparison of QSM MRI results, using the CSF reference mask, across the three populations studied. | **13** |
| **Supplementary Table 6:** Correlations, between iron concentration in regions of interest measured by QSM-MRI and Neuromelanin-MRI parameters in the SN. | **14** |
| **Supplementary Table 7:** Correlations between Neuromelanin variables and clinical characteristics in the HDGECs group. | **15** |
| **Supplementary Table 8:** Correlations, in the whole HDGECs group, between Neuromelanin variables and the neuropsychological tests. | **17** |
| **Supplementary Table 9:** Correlations between QSM variables and clinical characteristics in the HDGECs group.group, between QSM MRI variables and the neuropsychological tests. | **20** |
| **Supplementary Table 10:** Correlations, in the whole HDGECs group, between QSM MRI variables and the neuropsychological tests. | **22** |

**Supplementary Methods.**

**Clinical assessment:**

All participants completed a comprehensive battery of assessments encompassing clinical, motor, cognitive, and neuropsychiatric domains. This was performed on a single day in one sitting. The following clinical scales were administered to all participants: the Unified Huntington’s Disease Rating Scale Total Motor Score (UHDRS-TMS),^1^ UHDRS-Total Functional Capacity (TFC), UHDRS-Functional Assessment Scale (FAS), and the UHDRS-Independence Scale (IS). Motor function was assessed with the Physical Performance Test (PPT) and the Digital Finger tapping test (<https://appsto.re/us/ehHnA>). Behavioral symptoms were screened with the Problem Behavioral Assessment Short Version (PBA-s).^2^ Depressive symptomatology was assessed with the Beck Depression Inventory-II (BDI-II).^3^ Quality of life was evaluated with the HD Quality of Life scale (HDQoL).

A comprehensive paper-and-pencil battery of neuropsychological tests was administered to all participants. Cognitive screening was performed with the Montreal Cognitive Assessment (MoCA).^4^ Working memory was tested using the Digit Span Backwards - a subtest of the Wechsler Adult Intelligence Scale-IV; verbal fluency (phonemic and semantic),^5^ as language parameter, was tested with the Chi-Sigma-Alpha,^6^ and the Animals-Fruits-Objects tests, respectively. Attention, information processing speed, and cognitive flexibility were measured with the Trail Making Test (TMT, part A and B),^7^ by calculating the Ratio and the Difference of the TMT and using those scores for subsequent analyses. Divided attention, as an executive function parameter, was measured with the Symbol Digit Modalities Test (SDMT, written and oral forms).^8^ The Stroop Neuropsychological Screening Test^9^ was used to evaluate complex attention and inhibition of response. Verbal memory and learning was measured with the Hopkins Verbal Learning Test-Revised (HVLT-R)^10^ and visuospatial memory and learning with the Brief Visuospatial Memory Test-Revised (BVMT-R).^11^ Identity and emotion matching was measured with the two conditions of the Kinney’s Affect Matching Test: the Identity Matching Test (KIMT) and the Affect Matching Test (KAMT).^12^

**Neuromelanin-sensitive MRI analysis methods**

For the analysis of neuromelanin-sensitive MRI data, as a first step all images were transferred to a Linux workstation for analysis. The Neuromelanin-sensitive MRI analysis was performed by a neurologist with eight years of experience in neuroimaging analysis, on two separate days. Different ROIs were delineated for the calculation of the contrast-to-noise ratio (CNR) and the areas of the SN and LC.^13-15^ For the quantification of the high-signal regions in the SN and in the LC, the regions-of-interest (ROIs) were manually delineated twice on each subjects’ native space using Analyze Software, V.14.0 (Analyze, Biomedical Imaging Resources, Mayo Clinic, USA). The SN was identified as a hyperintense area placed dorsally to the cerebellar peduncles and ventrally to the red nucleus. This was identified on three consecutive slices. To account for variability and to avoid identifying the subthalamic nucleus which lies immediately rostrally to the SN, the middle slice was used for manual delineation ^16^. To calculate the SN intensity, two symmetric 2.4 mm squares were placed bilaterally corresponding to the medial and the lateral SN (**eFigure 1**). To calculate the reference intensity, two 4.0 mm squares were placed bilaterally on the cerebral peduncles to calculate the SN intensity. The LC was identified as the neuromelanin-hyperintense vertical structure placed in the posterior pons adjacent to the lateral floor of the fourth ventricle ^17^. A single slice corresponding to the rostral pons where the LC could be visually identified as point-like bilateral areas of hyperintensity bordering the fourth ventricle was used in all images for ROI delineation. To calculate the LC intensity, a 2.4 mm square was placed bilaterally over the hyperintense areas corresponding to the LC and a 5.6 mm reference region was placed over the middle of the brainstem (**eFigure 1**). This procedure was performed twice on the same slices for all images.

The ROIs were sampled, and the contrast-to-noise (CNR) ratio was calculated as (INT_ROI_-INT_REF_)/SD_REF_, where INT_ROI_ is the averaged intensity obtained from the SN and LC, respectively, INT_REF_ is the averaged intensity obtained from the SN and LC reference regions, respectively, and SD_REF_ is the standard deviation of the reference intensities.

As a second step, hyperintensity ROIs pertaining to the left and right SN and LC were manually drawn on the same slices previously considered for CNR calculation. These areas were then sampled on Analyze v.14 for calculation of the areas (**eFigure 2**). This was also done twice.

A two-way random Intraclass Correlation Coefficient was performed to assess reliability for both the CNR and the left and right SN and LC areas. The average measure showed good-to-excellent reliability with alpha values ranging from 0.782 to 0.917 (**eTable 1**). Then, the two CNRs values obtained from the two measurements were averaged to obtain a single CNR, which was used for analysis. To calculate the areas, the ROIs were merged using FMRIB Software Library (FSL) fslmaths utility ^18^ to obtain a final ROI composed of the intersecting voxels of measures #1 and #2. This ROI was sampled; and the left and right SN and LC areas were averaged to finally obtain a single value for analysis.

**QSM Analysis methods.**

Real and imaginary images for each echo were first converted to phase and magnitude images using mri_convert (https://github.com/rordenlab/spmScripts/blob/master/nii_complex2magPhase.m). The SEPIA toolbox (v1.2.2.6; https://sepia-documentation.readthedocs.io/en/latest/index.html), run in Matlab v2018b, which combines various QSM toolboxes, was used for QSM processing ^19^. For total field recovery and phase unwrapping, optimum weights ^20^ was applied for echo phase combination for temporal phase unwrapping of multi-echo data; and the Laplacian-based phase unwrapping method ^21^, using the MEDI toolbox within SEPIA, was performed for spatial phase unwrapping. The magnitude image at the first echo (TE = 4.5ms) was skull-stripped using FSL BET ^22^ to obtain a brain mask. The background field was removed using the V-SHARP method^23^. The QSM algorithm iterative LSQR^23^ was used to create a magnetic susceptibility Chimap, in parts per million (ppm). The brain mask was selected as the primary reference tissue following the 2023 Consensus recommendation ^24^. Analysis was also run using the CSF as the reference tissue, for comparison with previous studies, results using the CSF mask are provided in **eTable 5**. All susceptibility values are reported relative to the mean susceptibility value of the reference region.

For quantification of regional susceptibility, subcortical structural segmentation was performed using SEPIA analysis tool which employs non-linear registration using Advanced normalisation Tools (ANTs; https://github.com/ANTsX/ANTs) to bring the atlas into subjects GRE space. The multi-modal-fused magnetic susceptibility (MuSus-100) atlas was used to define regions of interest ^25^. Bias field correction on both the T1-weighted and GRE image was applied using N4BiasFieldCorrection ^26^. The T1-weighted image was skull-stripped using FSL BET ^22^ to obtain a brain mask, alongside the brain mask previously obtained for the GRE magnitude image at the first echo (TE = 4.5ms). The SEPIA analysis tool for the MuSus-100 atlas uses rigid-body transformation to co-register the GRE image and the T1-weighted image, then the Chimap is brought to T1-weighted space to create a hybrid image which is subsequently co-registered to the template hybrid image provided with the MuSus-100 atlas using nonlinear transformation. Finally, the derived transformation matrices were applied to bring the atlas labels into the subject’s GRE space. The fslstats utility ^18^ was used to extract susceptibility values (ppm) from the regions of interest from the MuSus-100 atlas. Regions of interest included: caudate, putamen, globus pallidus external, globus pallidus internal, SN pars compacta, SN pars reticulata, subthalamic nucleus, and red nucleus. Manual corrections on the caudate were applied in Analyze (v14.0) to improve atlas fitting around the caudate and avoid sampling into the ventricle.

**References:**

1. Unified Huntington's Disease Rating Scale: reliability and consistency. Huntington Study Group. *Mov Disord*. Mar 1996;11(2):136-42. doi:10.1002/mds.870110204

2. McNally G, Rickards H, Horton M, Craufurd D. Exploring the Validity of the Short Version of the Problem Behaviours Assessment (PBA-s) for Huntington's disease: A Rasch Analysis. *J Huntingtons Dis*. 2015;4(4):347-69. doi:10.3233/jhd-150164

3. Beck AT, Steer RA, Ball R, Ranieri W. Comparison of Beck Depression Inventories -IA and -II in psychiatric outpatients. *J Pers Assess*. Dec 1996;67(3):588-97. doi:10.1207/s15327752jpa6703_13

4. Konstantopoulos K, Vogazianos P. Montreal Cognitive Assessment in a Greek sample of patients with multiple sclerosis: A validation study. *Appl Neuropsychol Adult*. Jan-Feb 2021;28(1):48-52. doi:10.1080/23279095.2019.1588123

5. D. W. Wechsler Adult Intelligence Scale–Fourth Edition (WAIS–IV). Pearson Assessment.

6. Kosmidis MH, Vlahou CH, Panagiotaki P, Kiosseoglou G. The verbal fluency task in the Greek population: normative data, and clustering and switching strategies. *J Int Neuropsychol Soc*. Mar 2004;10(2):164-72. doi:10.1017/s1355617704102014

7. Zalonis I, Kararizou E, Triantafyllou NI, et al. A normative study of the trail making test A and B in Greek adults. *Clin Neuropsychol*. Sep 2008;22(5):842-50. doi:10.1080/13854040701629301

8. Messinis L, Bakirtzis C, Kosmidis MH, et al. Symbol Digit Modalities Test: Greek Normative Data for the Oral and Written Version and Discriminative Validity in Patients with Multiple Sclerosis. *Arch Clin Neuropsychol*. Jan 15 2021;36(1):117-125. doi:10.1093/arclin/acaa028

9. Zalonis I, Christidi F, Bonakis A, et al. The stroop effect in Greek healthy population: normative data for the Stroop Neuropsychological Screening Test. *Arch Clin Neuropsychol*. Feb 2009;24(1):81-8. doi:10.1093/arclin/acp011

10.  Liozidou, A., Fragkoulakis, M., & Zalonis, I. (2018). Hopkins Verbal Learning Test-Revised: Preliminary Greek normative data for research use. Dialogues in Clinical Neuroscience & Mental Health, 1(Suppl 1), 22. doi: 10.13140/RG.2.1.1986.1363

11. Arampatzi X, Margioti ES, Messinis L, et al. Development of robust normative data for the neuropsychological assessment of Greek older adults. *J Int Neuropsychol Soc*. Jul 2024;30(6):594-602. doi:10.1017/s1355617723011499

12. Kinney J.M. FBD, Pascualvaca D.M., Rodriguez D., Mirsky A.F. Comprehension of affect in children with pervasive developmental disorders: deficits in matching faces to cartoon situations. *Journal of the International Neuropsychological Society*. 1995;1:156-157.

13. Wang J, Li Y, Huang Z, et al. Neuromelanin-sensitive magnetic resonance imaging features of the substantia nigra and locus coeruleus in de novo Parkinson's disease and its phenotypes. *Eur J Neurol*. Jul 2018;25(7):949-e73. doi:10.1111/ene.13628

14. Wang X, Huang P, Haacke EM, et al. Locus coeruleus and substantia nigra neuromelanin magnetic resonance imaging differentiates Parkinson's disease and essential tremor. *Neuroimage Clin*. 2023;38:103420. doi:10.1016/j.nicl.2023.103420

15. Fabbri M, Reimão S, Carvalho M, et al. Substantia Nigra Neuromelanin as an Imaging Biomarker of Disease Progression in Parkinson's Disease. *J Parkinsons Dis*. 2017;7(3):491-501. doi:10.3233/jpd-171135

16. Wang L, Yan Y, Zhang L, Liu Y, Luo R, Chang Y. Substantia nigra neuromelanin magnetic resonance imaging in patients with different subtypes of Parkinson disease. *J Neural Transm (Vienna)*. Feb 2021;128(2):171-179. doi:10.1007/s00702-020-02295-8

17. Dahlström A, Fuxe K. Localization of monoamines in the lower brain stem. *Experientia*. Jul 15 1964;20(7):398-9. doi:10.1007/bf02147990

18. Jenkinson M, Beckmann CF, Behrens TE, Woolrich MW, Smith SM. FSL. *Neuroimage*. Aug 15 2012;62(2):782-90. doi:10.1016/j.neuroimage.2011.09.015

19. Chan KS, Marques JP. SEPIA-Susceptibility mapping pipeline tool for phase images. *Neuroimage*. Feb 15 2021;227:117611. doi:10.1016/j.neuroimage.2020.117611

20. Robinson SD, Bredies K, Khabipova D, Dymerska B, Marques JP, Schweser F. An illustrated comparison of processing methods for MR phase imaging and QSM: combining array coil signals and phase unwrapping. *NMR Biomed*. Apr 2017;30(4)doi:10.1002/nbm.3601

21. Schofield MA, Zhu Y. Fast phase unwrapping algorithm for interferometric applications. *Opt Lett*. Jul 15 2003;28(14):1194-6. doi:10.1364/ol.28.001194

22. Smith SM. Fast robust automated brain extraction. *Hum Brain Mapp*. Nov 2002;17(3):143-55. doi:10.1002/hbm.10062

23. Li W, Wu B, Liu C. Quantitative susceptibility mapping of human brain reflects spatial variation in tissue composition. *Neuroimage*. Apr 15 2011;55(4):1645-56. doi:10.1016/j.neuroimage.2010.11.088

24. Bilgic B, Costagli M, Chan KS, et al. Recommended Implementation of Quantitative Susceptibility Mapping for Clinical Research in The Brain: A Consensus of the ISMRM Electro-Magnetic Tissue Properties Study Group. *ArXiv*. Jul 5 2023;

25. He C, Guan X, Zhang W, et al. Quantitative susceptibility atlas construction in Montreal Neurological Institute space: towards histological-consistent iron-rich deep brain nucleus subregion identification. *Brain Struct Funct*. Jun 2023;228(5):1045-1067. doi:10.1007/s00429-022-02547-1

26. Tustison NJ, Avants BB, Cook PA, et al. N4ITK: improved N3 bias correction. *IEEE Trans Med Imaging*. Jun 2010;29(6):1310-20. doi:10.1109/tmi.2010.2046908

**Supplementary Figure 1:** Description of the methods used to calculate the contrast-to-noise ratio (CNR) in neuromelanin MRI images. Squared areas were placed on the left and right loci coerulei, the left and right substantia nigra (SN) and the reference regions in two separate sessions (measure #1 and measure #2). The intensity values were extracted and an intraclass correlation coefficient (ICC) was performed. The values from the two measurements were averaged to obtain a single SN CNR and LC CNR to be used for analysis.


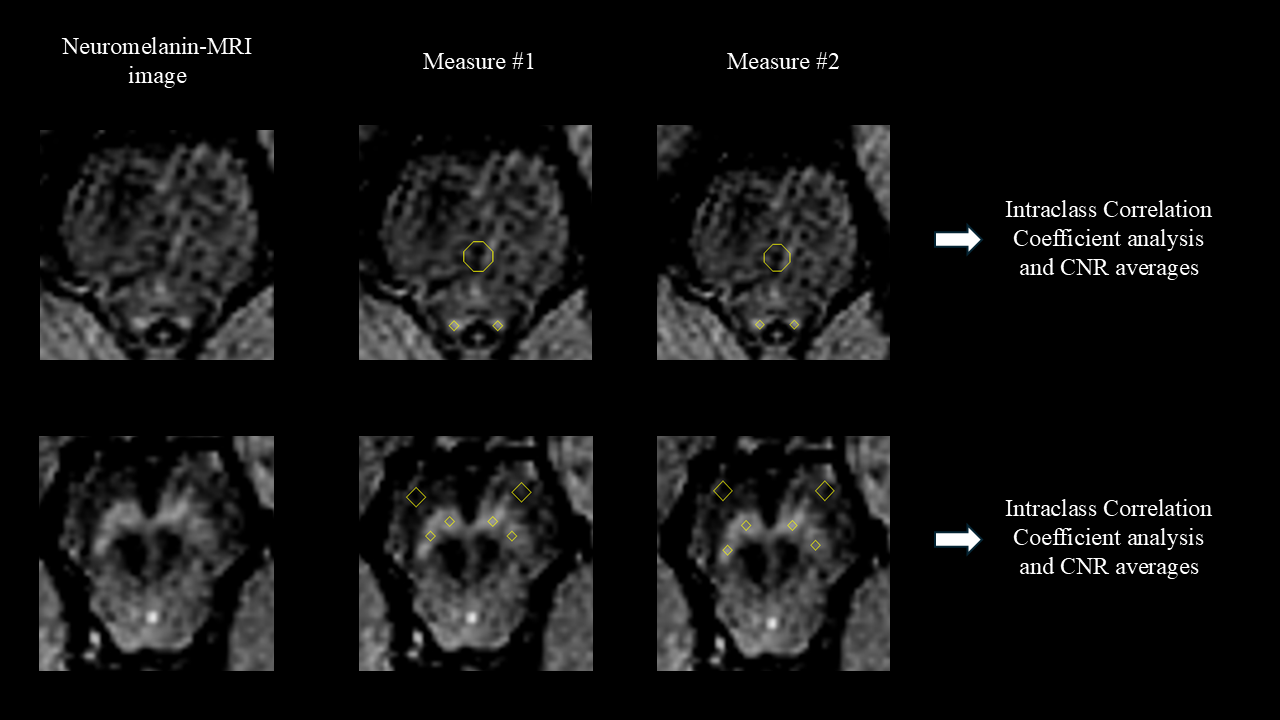


**Supplementary Figure 2:** Description of the method used to sample the SN and LC areas in neuromelanin MRI images. The left and right LC and SN areas were manually drawn on a single slice in two separate sessions (measure #1 and measure #2). Their areas were sampled and an intraclass correlation coefficient (ICC) was performed. The left and right LC and SN areas were then averaged to obtain a single SN area and a single LC area that were used for analysis.

**
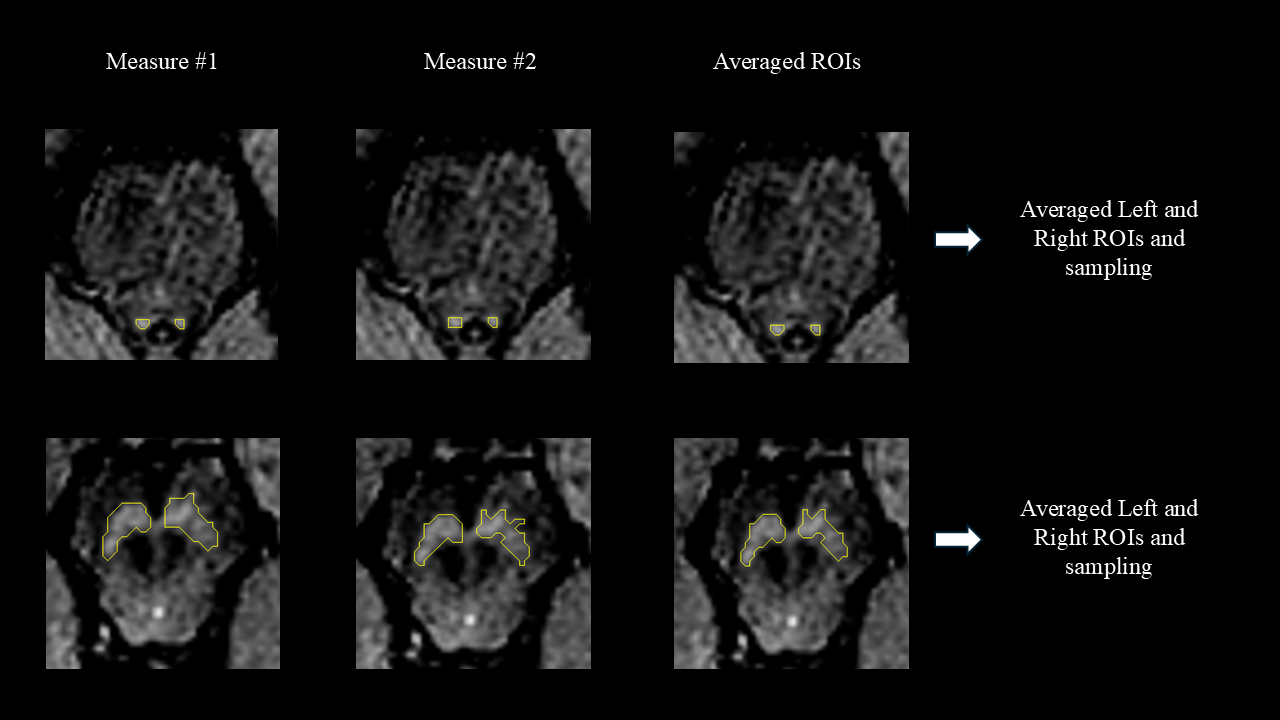
**

| **Supplementary Table 1:** Intraclass Correlation Coefficient between the measures #1 and #2 of Neuromelanin regions of interest. | | |
| --- | --- | --- |
| **Region** | **ICC [95% CI]** | ***p* value** |
| **SN Area Left** | 0.898 [0.816 – 0.943] | <0.001 |
| **SN Area Right** | 0.851 [0.726 – 0.918] | <0.001 |
| **SN CNR** | 0.917 [0.852 – 0.954] | <0.001 |
| **LC Area Left** | 0.784 [0.608 – 0.881] | <0.001 |
| **LC Area Right** | 0.782 [0.608 – 0.879] | <0.001 |
| **LC CNR** | 0.880 [0.786 – 0.933] | <0.001 |
| **Abbreviations:** CNR: contrast-to-noise ratio; LC: locus coeruleus; SN: substantia nigra. | | |

| **Supplementary Table 2.** Differences in neuropsychological tests between the three populations studied. | | | | | | |
| --- | --- | --- | --- | --- | --- | --- |
| **Item** | **HC (n=25)** | **Premanifest HDGECs (n=11)** | **Manifest HDGECs (n=14)** | ***p* HC vs premanifest HDGECs** | ***p* HC vs manifest HDGECs** | ***p* premanifest HDGECs vs manifest HDGECs** |
| **MoCA** | 27.92 ± 1.29 | 24.72 ± 2.72 | 17.21 ± 4.93 | 0.003 | <0.001 | <0.001 |
| **Digit Span backwards** | 9.28 ± 1.99 | 7.55 ± 1.97 | 4.71 ± 1.86 | 0.012 | <0.001 | 0.001 |
| **Verbal Fluency Phonemic** | 45.28 ± 10.06 | 32.73 ± 11.92 | 9.64 ± 6.83 | 0.004 | <0.001 | <0.001 |
| **Verbal Fluency Semantic** | 63.04 ± 10.06 | 53.64 ± 7.63 | 25.36 ± 8.02 | 0.005 | <0.001 | <0.001 |
| **TMT ratio** | 1.87 ± 0.42 | 2.23 ± 0.54 | 3.1 ± 1.37 | 0.063 | <0.001 | 0.052 |
| **TMT difference** | 25.32 ± 14.48 | 50.55 ± 28.13 | 157.64 ± 70.32 | 0.001 | <0.001 | <0.001 |
| **SDMT – Oral** | 63.28 ± 12.20 | 48.73 ± 11.48 | 19.57 ± 7.06 | 0.003 | <0.001 | <0.001 |
| **SDMT - Written** | 54.48 ± 9.57 | 39.91 ± 9.67 | 16.36 ± 5.60 | <0.001 | <0.001 | <0.001 |
| **Stroop colour-word interference** | 106.04 ± 9.14 | 91.09 ± 20.36 | 33.29 ± 21.90 | 0.045 | <0.001 | <0.001 |
| **HVLT-R total** | 25.48 ± 3.36 | 21.73 ± 3.98 | 11.00 ± 3.66 | 0.014 | <0.001 | <0.001 |
| **HVLT-R delayed recall** | 8.56 ± 1.64 | 4.55 ± 4.25 | 0.71 ± 1.14 | 0.009 | <0.001 | 0.038 |
| **HVLT-R discrimination index** | 11.60 ± 0.65 | 8.27 ± 2.37 | 3.57 ± 3.63 | <0.001 | <0.001 | <0.001 |
| **HVLT-R % retained** | 82.09 ± 12.77 | 47.30 ± 42.93 | 17.35 ± 26.13 | 0.018 | <0.001 | 0.075 |
| **BVMT-R total** | 30.28 ± 3.82 | 22.09 ± 6.02 | 9.50 ± 6.05 | <0.001 | <0.001 | <0.001 |
| **BVMT-R delayed recall** | 11.16 ± 1.07 | 8.91 ± 2.59 | 3.64 ± 2.53 | 0.004 | <0.001 | <0.001 |
| **BVMT-R discrimination index** | 5.96 ± 0.20 | 5.82 ± 0.40 | 3.21 ± 2.29 | 0.520 | <0.001 | 0.005 |
| **BVMT-R % retained** | 96.21 ± 7.01 | 99.46 ± 10.97 | 78.17 ± 40.37 | 0.342 | 0.133 | 0.104 |
| **KIMT** | 29.24 ± 1.01 | 27.00 ± 1.79 | 22.07 ± 5.44 | <0.001 | <0.001 | 0.009 |
| **KAMT** | 29.20 ± 1.15 | 27.36 ± 2.34 | 20.14 ± 8.04 | 0.011 | <0.001 | 0.009 |
| **Abbreviations:** BVMT-R: Brief Visuospatial Memory Test-Revised; HDGECs: Huntington’s Disease gene expansion carriers; HC: Healthy Controls; HVLT-R: Hopkins Verbal Learning Test – Revised; KAMT: Kinney’s Affect Matching Test; KIMT: Kinney’s Identity Matching Test; MoCA: Montreal Cognitive Assessment; TMT: Trail Making Test; Student’s *t*-test for parametric variables, or Mann Whitney U test for non-parametric variables. | | | | | | |

| **Supplementary Table 3:** Neuromelanin MRI results across the three populations studied. | | | | | | | |
| --- | --- | --- | --- | --- | --- | --- | --- |
|  | **HC (n=23)** | **Premanifest HDGECs (n=10)** | **Manifest HDGECs (n=14)** | **MANCOVA** | ***p* (HC vs premanifest HDGECs)** | ***p* (HC vs manifest HDGECs)** | ***p* (premanifest HDGECs vs manifest HDGECs)** |
| **SN Area (mm^2^)** | 42.47 ± 4.95 | 38.49 ± 5.92 | 32.21 ± 4.87 | F=15.785 *p*<0.001 | 0.149 | <0.001 | 0.023 |
| **SN CNR** | 4.27 ± 1.45 | 4.41 ± 1.70 | 3.19 ± 1.23 | F=1.998 *p*=0.150 | 1.00 | 0.238 | 0.293 |
| **LC Area (mm^2^)** | 5.06 ± 0.65 | 4.65 ± 0.43 | 3.58 ± 0.92 | F=17.615 *p*<0.001 | 0.351 | <0.001 | 0.004 |
| **LC CNR** | 4.83 ± 1.65 | 3.85 ± 1.00 | 2.95 ± 0.92 | F=11.200 *p*<0.001 | 0.215 | <0.001 | 0.095 |
| **Abbreviations:** HC: Healthy controls; HDGECs: Huntington’s disease gene expansion carriers; LC: Locus Coeruleus; SN: Substantia Nigra. Multivariate analysis of covariance, with age and sex as covariates, and Bonferroni correction (*p*=0.0125). | | | | | | | |

| **Supplementary Table 4:** Comparison of QSM MRI results, using the whole brain reference mask, across the three populations studied. | | | | | | | |
| --- | --- | --- | --- | --- | --- | --- | --- |
|  | **HC (n=25)** | **Premanifest HDGECs (n=10)** | **Manifest HDGECs (n=13)** | **MANCOVA** | ***p* (HC vs premanifest HDGECs)** | ***p* (HC vs manifest HDGECs)** | ***p* (premanifest HDGECs vs manifest HDGECs)** |
| **Red Nucleus** | 0.068 ± 0.019 | 0.061 ± 0.018 | 0.090 ± 0.030 | F=4.006 p=0.025 | 1.00 | 0.057 | 0.04 |
| **SN pars compacta** | 0.069 ± 0.011 | 0.072 ± 0.023 | 0.073 ± 0.024 | F=0.155 p=0.857 | 1.00 | 1.00 | 1.00 |
| **SN pars reticulata** | 0.101 ± 0.017 | 0.109 ± 0.030 | 0.132 ± 0.040 | F=3.919 p=0.027 | 1.00 | 0.023 | 0.301 |
| **Subthalamic nucleus** | 0.082 ± 0.019 | 0.082 ± 0.026 | 0.116 ± 0.024 | F=8.693 p<0.001 | 1.00 | <0.001 | 0.01 |
| **Internal pallidum** | 0.076 ± 0.012 | 0.094 ± 0.025 | 0.149 ± 0.023 | F=27.483 p<0.001 | 0.683 | <0.001 | <0.001 |
| **External pallidum** | 0.072 ± 0.013 | 0.084 ± 0.022 | 0.127 ± 0.034 | F=53.857 p<0.001 | 0.059 | <0.001 | <0.001 |
| **Ventral pallidum** | 0.098 ± 0.029 | 0.084 ± 0.029 | 0.127 ± 0.034 | F=3.266 p=0.048 | 0.712 | 0.237 | 0.047 |
| **Caudate** | 0.032 ± 0.005 | 0.037 ± 0.010 | 0.054 ± 0.011 | F=25.956 p<0.001 | 0.251 | <0.001 | <0.001 |
| **Putamen** | 0.024 ± 0.007 | 0.030 ± 0.017 | 0.072 ± 0.027 | F=36.888 p<0.001 | 0.416 | <0.001 | <0.001 |
| **Accumbens** | -0.0002 ± 0.016 | 0.002 ± 0.019 | 0.016 ± 0.013 | F=3.379 p=0.043 | 1.00 | 0.038 | 0.379 |
| **Abbreviations:** HC: Healthy controls; HDGECs: Huntington’s disease gene expansion carriers; Multivariate analysis of covariance, with age and sex as covariates, and Bonferroni correction (*p*=0.005). | | | | | | | |

| **Supplementary Table 5:** Comparison of QSM MRI results, using CSF reference mask, across the three populations studied. | | | | | | | |
| --- | --- | --- | --- | --- | --- | --- | --- |
|  | **HC (n=25)** | **Premanifest HDGECs (n=10)** | **Manifest HDGECs (n=13)** | **MANCOVA** | ***p* (HC vs premanifest HDGECs)** | ***p* (HC vs manifest HDGECs)** | ***p* (premanifest HDGECs vs manifest HDGECs)** |
| **Red nucleus** | 0.064 ± 0.019 | 0.052 ± 0.019 | 0.088 ± 0.031 | F=5.981 p=0.005 | 0.689 | 0.028 | 0.006 |
| **SN pars compacta** | 0.065 ± 0.016 | 0.063 ± 0.024 | 0.071 ± 0.025 | F=0.355 p=0.703 | 1.00 | 1.00 | 1.00 |
| **SN pars reticulata** | 0.098 ± 0.020 | 0.100 ± 0.028 | 0.130 ± 0.041 | F=4.246 p=0.021 | 1.00 | 0.021 | 0.109 |
| **Subthalamic nucleus** | 0.078 ± 0.022 | 0.073 ± 0.023 | 0.114 ± 0.025 | F=9.675 p<0.001 | 1.00 | <0.001 | 0.002 |
| **External pallidum** | 0.073 ± 0.014 | 0.085 ± 0.025 | 0.146 ± 0.024 | F=52.630 p<0.001 | 0.345 | <0.001 | <0.001 |
| **Internal pallidum** | 0.069 ± 0.014 | 0.075 ± 0.023 | 0.125 ± 0.034 | F=28.850 p<0.001 | 1.00 | <0.001 | <0.001 |
| **Ventral pallidum** | 0.095 ± 0.030 | 0.075 ± 0.031 | 0.117 ± 0.034 | F=4.420 p=0.018 | 0.360 | 0.180 | 0.015 |
| **Caudate** | 0.029 ± 0.010 | 0.028 ± 0.015 | 0.050 ± 0.016 | F=11.558 p<0.001 | 1.00 | <0.001 | 0.002 |
| **Putamen** | 0.020 ± 0.009 | 0.021 ± 0.021 | 0.070 ± 0.029 | F=31.877 p<0.001 | 1.00 | <0.001 | <0.001 |
| **Accumbens** | -0.004 ± 0.016 | -0.007 ± 0.023 | 0.014 ± 0.015 | F=4.492 p=0.017 | 1.00 | 0.024 | 0.048 |
| **Abbreviations:** CSF: Cerebrospinal fluid; GPe: external globus pallidus; GPi: internal globus pallidus; HC: Healthy controls; HDGECs: Huntington’s disease gene expansion carriers; SNc: Substantia Nigra pars compacta; SNr: Substantia Nigra pars reticulata. Multivariate analysis of covariance, with age and sex as covariates, and Bonferroni correction. | | | | | | | |

| **Supplementary Table 6:** Correlations, between iron concentration in regions of interest measured by QSM-MRI and Neuromelanin-MRI parameters in the SN. | | | | |
| --- | --- | --- | --- | --- |
|  | **All HDGECs** | | **Manifest HDGECs only** | |
|  | **Neuromelanin SN Area** | **Neuromelanin SN CNR** | **Neuromelanin SN Area** | **Neuromelanin SN CNR** |
| **QSM Caudate** | Rho: -0.329 p=0.146  FDR: p=0.195 | Rho: -0.560 p=0.008  FDR: p=0.032 | Rho: 0.120 p=0.725  FDR: p=0.949 | Rho: -0.641 p=0.034  FDR: p=0.068 |
| **QSM Putamen** | Rho: -0.401 p=0.071  FDR: p=0.195 | Rho: -0.519  p=0.016  FDR: p=0.032 | Rho: -0.022 p=0.949  FDR: p=0.949 | Rho: -0.735  p=0.01  FDR: p=0.04 |
| **QSM External Pallidum** | Rho: -0.334 p=0.138  FDR: p=0.195 | Rho: -0.343 p=0.128  FDR: p=0.171 | Rho: 0.171 p=0.614  FDR: p=0.949 | Rho: -0.106 p=0.757  FDR: p=0.757 |
| **QSM Internal Pallidum** | Rho: -0.170 p=0.461  FDR: p=0.461 | Rho: -0.306 p=0.177  FDR: p=0.177 | Rho: 0.311 p=0.352  FDR: p=0.949 | Rho: 0.106 p=0.757  FDR: p=0.757 |
| **Abbreviations:** CNR: Contrast to Noise Ratio; QSM: Quantitative Susceptibility Mapping; SN: Substantia Nigra. Pearson’s partial correlation analysis controlling for age and sex. In red are the significant *p* values after FDR correction. | | | | |

| **Supplementary Table 7:** Correlations between Neuromelanin variables and clinical characteristics in the HDGECs group. | | | |
| --- | --- | --- | --- |
|  | **SN Area** | **LC Area** | **LC CNR** |
| **Disease Duration*** | Rho: -0.296 p=0.350  FDR: p=0.350 | Rho: -0.402 p=0.195  FDR: p=0.211 | Rho: -0.401 p=0.197  FDR: p=0.375 |
| **CAG long allele** | Rho: -0.394 p=0.063  FDR: p=0.136 | Rho: -0.347 p=0.105  FDR: p=0.143 | Rho: -0.097 p=0.683  FDR: p=0.885 |
| **Disease Burden Score** | Rho: -0.335 p=0.148  FDR: p=0.175 | Rho: -0.272 p=0.247  FDR: p=0.247 | Rho: -0.131 p=0.583  FDR: p=0.842 |
| **CAP Score** | Rho: -0.372 p=0.073  FDR: p=0.136 | Rho: -0.298 p=0.157  FDR: p=0.185 | Rho: -0.016 p=0.941  FDR: p=0.943 |
| **UHDRS-TMS*** | Rho: -0.457 p=0.135  FDR: p=0.175 | Rho: -0.514 p=0.087  FDR: p=0.141 | Rho: -0.211 p=0.511  FDR: p=0.830 |
| **UHDRS-TFC*** | Rho: 0.423 p=0.171  FDR: p=0.185 | Rho: 0.536 p=0.073  FDR: p=0.136 | Rho: -0.023 p=0.943  FDR: p=0.943 |
| **HDQoL** | Rho: -0.586 p=0.007  FDR: p=0.059 | Rho: -0.664 p=0.001  FDR: p=0.013 | Rho: -0.431 p=0.058  FDR: p=0.189 |
| **PPT*** | Rho: 0.461 p=0.132  FDR: p=0.175 | Rho: 0.548 p=0.065  FDR: p=0.136 | Rho: -0.104 p=0.749  FDR: p=0.885 |
| **PBA** | Rho: -0.568 p=0.009  FDR: p=0.059 | Rho: -0.607 p=0.005  FDR: p=0.021 | Rho: -0.485 p=0.03  FDR: p=0.189 |
| **Finger Tapping R** | Rho: 0.470 p=0.036  FDR: p=0.124 | Rho: 0.656 p=0.002  FDR: p=0.013 | Rho: 0.450 p=0.047  FDR: p=0.189 |
| **Finger Tapping L** | Rho: 0.436 p=0.055  FDR: p=0.136 | Rho: 0.576 p=0.008  FDR: p=0.026 | Rho: 0.298 p=0.202  FDR: p=0.375 |
| **BDI-II** | Rho: -0.467 p=0.038  FDR: p=0.124 | Rho: -0.368 p=0.110  FDR: p=0.143 | Rho: -0.365 p=0.114  FDR: p=0.296 |
| **Plasma NfL levels** | Rho: -0.342 p=0.140  FDR: p=0.175 | Rho: -0.553 p=0.011  FDR: p=0.029 | Rho: -0.472 p=0.036  FDR: p=0.189 |
| **Abbreviations:** BDI-II: Beck Depression Inventory II; CAG: cytosine-adenine-guanine; CAP: CAG-Age-Product; CNR: Contrast-to-Noise ratio; LC: Locus coeruleus; NfL: Neurofilament light chain; PBA: Problem Behaviour Assessment; PPT: Physical Performance Test; SN: Substantia Nigra; TFC: Total Functional Capacity; TMS: Total Motor Score; UHDRS: Unified Huntington’s Disease Rating Scale. Pearson’s partial correlation analysis controlling for age and sex. The CAP score is a function of age so Pearson’s partial correlation controlling for sex only was used. All analyses performed on the whole HDGECs cohort apart from * = in manifest HDGECs only. In red are the significant *p* values after FDR correction. | | | |

| **Supplementary Table 8:** Correlations, in the whole HDGECs group, between Neuromelanin variables and the neuropsychological tests. | | | |
| --- | --- | --- | --- |
|  | **SN Area** | **LC Area** | **LC CNR** |
| **MoCA** | Rho: 0.314 p=0.155  FDR: p=0.200 | Rho: 0.185 p=0.410  FDR: p=0.487 | Rho: 0.075 p=0.741  FDR: p=0.782 |
| **Digit Span backwards** | Rho: 0.475 p=0.025  FDR: p=0.062 | Rho: 0.318 p=0.149  FDR: p=0.242 | Rho: 0.348 p=0.112  FDR: p=0.355 |
| **Verbal Fluency Phonemic** | Rho: 0.441 p=0.04  FDR: p=0.084 | Rho: 0.492 p=0.02  FDR: p=0.063 | Rho: 0.441 p=0.04  FDR: p=0.355 |
| **Verbal Fluency Semantic** | Rho: 0.534 p=0.01  FDR: p=0.062 | Rho: 0.584 p=0.004  FDR: p=0.063 | Rho: 0.378 p=0.083  FDR: p=0.355 |
| **TMT ratio** | Rho: -0.081 p=0.720  FDR: p=0.723 | Rho: -0.315 p=0.153  FDR: p=0.242 | Rho: -0.131 p=0.561  FDR: p=0.627 |
| **TMT difference** | Rho: -0.285 p=0.199  FDR: p=0.236 | Rho: -0.460 p=0.031  FDR: p=0.084 | Rho: -0.189 p=0.401  FDR: p=0.552 |
| **SDMT Oral** | Rho: 0.472 p=0.027  FDR: p=0.062 | Rho: 0.503 p=0.017  FDR: p=0.063 | Rho: 0.365 p=0.094  FDR: p=0.355 |
| **SDMT Written** | Rho: 0.562 p=0.007  FDR: p=0.062 | Rho: 0.435 p=0.043  FDR: p=0.102 | Rho: 0.408 p=0.059  FDR: p=0.355 |
| **Stroop colour-word interference** | Rho: 0.523 p=0.013  FDR: p=0.062 | Rho: 0.555 p=0.007  FDR: p=0.063 | Rho: 0.289 p=0.192  FDR: p=0.511 |
| **HVLT-R Immediate recall** | Rho: 0.535 p=0.01  FDR: p=0.062 | Rho: 0.522 p=0.013  FDR: p=0.063 | Rho: 0.367 p=0.093  FDR: p=0.355 |
| **HVLT-R delayed** | Rho: 0.324 p=0.141  FDR: p=0.20 | Rho: 0.213 p=0.341  FDR: p=0.432 | Rho: 0.269 p=0.225  FDR: p=0.511 |
| **HVLT-R discrimination index** | Rho: 0.316 p=0.152  FDR: p=0.20 | Rho: 0.376 p=0.084  FDR: p=0.160 | Rho: 0.196 p=0.381  FDR: p=0.552 |
| **HVLT-R % retained** | Rho: 0.157 p=0.485  FDR: p=0.542 | Rho: -0.056 p=0.804  FDR: p=0.804 | Rho: 0.167 p=0.457  FDR: p=0.579 |
| **BVMT-R immediate** | Rho: 0.360 p=0.10  FDR: p=0.20 | Rho: 0.221 p=0.322  FDR: p=0.432 | Rho: 0.186 p=0.407  FDR: p=0.552 |
| **BVMT-R delayed** | Rho: 0.312 p=0.158  FDR: p=0.20 | Rho: 0.170 p=0.45  FDR: p=0.503 | Rho: 0.189 p=0.401  FDR: p=0.552 |
| **BVMT-R discrimination index** | Rho: 0.422 p=0.05  FDR: p=0.095 | Rho: 0.240 p=0.281  FDR: p=0.411 | Rho: 0.153 p=0.496  FDR: p=0.589 |
| **BVMT-R % retained** | Rho: 0.080 p=0.723  FDR: p=0.723 | Rho: 0.059 p=0.793  FDR: p=0.804 | Rho: 0.036 p=0.875  FDR: p=0.875 |
| **KIMT** | Rho: 0.492 p=0.02  FDR: p=0.062 | Rho: 0.402 p=0.064  FDR: p=0.135 | Rho: 0.239 p=0.284  FDR: p=0.552 |
| **KAMT** | Rho: 0.472 p=0.027  FDR: p=0.062 | Rho: 0.516 p=0.014  FDR: p=0.063 | Rho: 0.260 p=0.242  FDR: p=0.511 |
| **Abbreviations:** BVMT-R: Brief Visuospatial Memory Test-Revised; HDGECs: Huntington’s Disease gene expansion carriers; HC: Healthy Controls; HVLT-R: Hopkins Verbal Learning Test – Revised; KAMT: Kinney’s Affect Matching Test; KIMT: Kinney’s Identity Matching Test; MoCA: Montreal Cognitive Assessment; TMT: Trail Making Test. Pearson’s partial correlation analysis controlling for age and sex. In red are the significant *p* values after FDR correction. | | | |

| **Supplementary Table 9:** Correlations between QSM variables and clinical characteristics in the HDGECs group.group, between QSM MRI variables and the neuropsychological tests. | | | | | |
| --- | --- | --- | --- | --- | --- |
|  | **Caudate QSM** | **Putamen QSM** | **External pallidum QSM** | **Internal pallidum QSM** | **Subthalamic Nucleus QSM** |
| **Disease Duration*** | Rho: 0.306 p=0.359  FDR: p=0.467 | Rho: 0.538 p=0.088  FDR: p=0.114 | Rho: 0.062 p=0.856  FDR: p=0.951 | Rho: -0.360 p=0.276  FDR: p=0.359 | Rho: 0.139 p=0.684  FDR: p=0.796 |
| **CAG long allele** | Rho: 0.421 p=0.065  FDR: p=0.094 | Rho: 0.432  p=0.057  FDR: p=0.082 | Rho: 0.679 p<0.001  FDR: p=0.002 | Rho: 0.569 p=0.009  FDR: p=0.036 | Rho: 0.255 p=0.278  FDR: p=0.402 |
| **Disease Burden Score** | Rho: 0.452 p=0.045  FDR: p=0.073 | Rho: 0.469 p=0.037  FDR: p=0.06 | Rho: 0.677 p=0.001  FDR: p=0.002 | Rho: 0.553 p=0.011  FDR: p=0.036 | Rho: 0.266 p=0.256  FDR: p=0.402 |
| **CAP Score** | Rho: 0.551 p=0.008  FDR: p=0.026 | Rho: 0.583 p=0.004  FDR: p=0.01 | Rho: 0.707 p<0.001  FDR: p=0.002 | Rho: 0.463 p=0.03  *FDR: p=0.05* | Rho: 0.446 p=0.037  FDR: p=0.149 |
| **UHDRS-TMS*** | Rho: 0.256 p=0.447  FDR: p=0.528 | Rho: 0.226 p=0.505  FDR: p=0.597 | Rho: 0.177 p=0.603  FDR: p=0.784 | Rho: 0.149 p=0.662  FDR: p=0.782 | Rho: 0.008 p=0.982  FDR: p=0.982 |
| **UHDRS-TFC*** | Rho: 0.030 p=0.930  FDR: p=0.974 | Rho: 0.073 p=0.83  FDR: p=0.901 | Rho: -0.052 p=0.878  FDR: p=0.951 | Rho: -0.027 p=0.937  FDR: p=0.937 | Rho: 0.116 p=0.735  FDR: p=0.796 |
| **HDQoL** | Rho: 0.516 p=0.02  FDR: p=0.043 | Rho: 0.574 p=0.008  FDR: p=0.017 | Rho: 0.663 p=0.001  FDR: p=0.002 | Rho: 0.535 p=0.015  *FDR: p=0.05* | Rho: 0.406 p=0.075  FDR: p=0.149 |
| **PPT*** | Rho: 0.011 p=0.974  FDR: p=0.974 | Rho: 0.027 p=0.938  FDR: p=0.938 | Rho: 0.013 p=0.970  FDR: p=0.970 | Rho: 0.073 p=0.831  FDR: p=0.900 | Rho: 0.136 p=0.689  FDR: p=0.796 |
| **PBA** | Rho: 0.618 p=0.004  FDR: p=0.026 | Rho: 0.643 p=0.002  FDR: p=0.007 | Rho: 0.726 p<0.001  FDR: p=0.002 | Rho: 0.590 p=0.006  FDR: p=0.036 | Rho: 0.448 p=0.048  FDR: p=0.149 |
| **Finger Tapping Right** | Rho: -0.552 p=0.012  FDR: p=0.031 | Rho: -0.690  p<0.001  FDR: p=0.003 | Rho: -0.644 p=0.002  FDR: p=0.003 | Rho: -0.508 p=0.022  *FDR: p=0.05* | Rho: -0.429 p=0.059  FDR: p=0.149 |
| **Finger Tapping Left** | Rho: -0.583 p=0.007  FDR: p=0.026 | Rho: -0.697  p<0.001  FDR: p=0.003 | Rho: -0.682 p<0.001  FDR: p=0.002 | Rho: -0.489 p=0.029  *FDR: p=0.05* | Rho: -0.401 p=0.08  FDR: p=0.149 |
| **BDI-II** | Rho: 0.466 p=0.038  FDR: p=0.071 | Rho: 0.487 p=0.029  FDR: p=0.054 | Rho: 0.577 p=0.008  FDR: p=0.012 | Rho: 0.441 p=0.052  FDR: p=0.075 | Rho: 0.471 p=0.036  FDR: p=0.149 |
| **Plasma NfL levels** | Rho: 0.694 p<0.001  FDR: p=0.026 | Rho: 0.689 p<0.001  FDR: p=0.003 | Rho: 0.664 p=0.001  FDR: p=0.002 | Rho: 0.578 p=0.008  FDR: p=0.036 | Rho: 0.438 p=0.053  FDR: p=0.149 |
| **Abbreviations:** BDI-II: Beck Depression Inventory II; CAG: cytosine-adenine-guanine; CAP: CAG-Age-Product; NfL: Neurofilament light chain; PBA: Problem Behaviour Assessment; PPT: Physical Performance Test; QSM: Quantitative susceptibility Mapping; TFC: Total Functional Capacity; TMS: Total Motor Score; UHDRS: Unified Huntington’s Disease Rating Scale. Pearson’s partial correlation analysis controlling for age and sex. The CAP score is a function of age so Pearson’s partial correlation analysis controlling for sex only was used. All analyses performed on the whole HDGECs cohort apart from * = in manifest HDGECs only. In red are the significant *p* values after FDR correction. | | | | | |

| **Supplementary Table 10:** Correlations, in the whole HDGECs group, between QSM MRI variables and the neuropsychological tests. | | | | | |
| --- | --- | --- | --- | --- | --- |
|  | **Caudate QSM** | **Putamen QSM** | **External pallidum QSM** | **Internal pallidum QSM** | **Subthalamic Nucleus QSM** |
| **MoCA** | Rho: -0.209 p=0.364  FDR: p=0.444 | Rho: -0.178 p=0.441  FDR: p=0.524 | Rho: -0.451 p=0.04  FDR: p=0.047 | Rho: -0.477 p=0.029  FDR: p=0.037 | Rho: -0.014 p=0.950  FDR: p=0.950 |
| **Digit Span backwards** | Rho: -0.436 p=0.048  FDR: p=0.101 | Rho: -0.491 p=0.024  FDR: p=0.057 | Rho: -0.590 p=0.005  FDR: p=0.009 | Rho: -0.456 p=0.038  FDR: p=0.045 | Rho: -0.146 p=0.527  FDR: p=0.589 |
| **Verbal fluency Phonemic** | Rho: -0.532 p=0.013  FDR: p=0.031 | Rho: -0.546 p=0.01  FDR: p=0.032 | Rho: -0.662 p=0.001  FDR: p=0.003 | Rho: -0.611 p=0.003  FDR: p=0.006 | Rho: -0.450 p=0.041  FDR: p=0.190 |
| **Verbal Fluency Semantic** | Rho: -0.619 p=0.003  FDR: p=0.013 | Rho: -0.663 p=0.001  FDR: p=0.006 | Rho: -0.731 p<0.001  FDR: p=0.001 | Rho:-0.640 p=0.002  FDR: p=0.006 | Rho: -0.427 p=0.053  FDR: p=0.190 |
| **TMT ratio** | Rho: 0.005 p=0.984  FDR: p=0.984 | Rho: 0.083 p=0.720  FDR: p=0.760 | Rho: 0.209 p=0.364  FDR: p=0.364 | Rho: 0.284 p=0.212  FDR: p=0.214 | Rho: 0.088 p=0.705  FDR: p=0.744 |
| **TMT difference** | Rho: 0.320 p=0.157  FDR: p=0.229 | Rho: 0.345 p=0.125  FDR: p=0.216 | Rho: 0.460 p=0.036  FDR: p=0.047 | Rho: 0.489 p=0.025  FDR: p=0.034 | Rho: 0.205 p=0.373  FDR: p=0.457 |
| **SDMT Oral** | Rho: -0.693 p<0.001  FDR: p=0.004 | Rho: -0.707  p<0.001  FDR: p=0.006 | Rho: -0.735 p<0.001  FDR: p=0.001 | Rho: -0.620 p=0.003  FDR: p=0.006 | Rho: -0.378 p=0.091  FDR: p=0.190 |
| **SDMT Written** | Rho: -0.677 p<0.001  FDR: p=0.004 | Rho: -0.649  p=0.001  FDR: p=0.006 | Rho: -0.698 p<0.001  FDR: p=0.002 | Rho: -0.576 p=0.006  FDR: p=0.011 | Rho: -0.411 p=0.064  FDR: p=0.190 |
| **Stroop colour-word interference** | Rho: -0.544 p=0.011  FDR: p=0.03 | Rho: -0.593 p=0.005  FDR: p=0.019 | Rho: -0.730 p<0.001  FDR: p=0.001 | Rho: -0.651 p=0.001  FDR: p=0.006 | Rho: -0.378 p=0.091  FDR: p=0.190 |
| **HVLT-R Immediate recall** | Rho: -0.597 p=0.004  FDR: p=0.013 | Rho: -0.594 p=0.005  FDR: p=0.019 | Rho: -0.791 p<0.001  FDR: p<0.001 | Rho: -0.689 p<0.001  FDR: p=0.006 | Rho: -0.417 p=0.06  FDR: p=0.190 |
| **HVLT-R delayed** | Rho: -0.682 p<0.001  FDR: p=0.004 | Rho: -0.512 p=0.018  FDR: p=0.049 | Rho: -0.693 p<0.001  FDR: p=0.001 | Rho: -0.672 p<0.001  FDR: p=0.006 | Rho: -0.395 p=0.076  FDR: p=0.190 |
| **HVLT-R discrimination index** | Rho: -0.385 p=0.085  FDR: p=0.162 | Rho: -0.460 p=0.036  FDR: p=0.076 | Rho: -0.617 p=0.003  FDR: p=0.007 | Rho: -0.529 p=0.014  FDR: p=0.022 | Rho: -0.476 p=0.029  FDR: p=0.190 |
| **HVLT-R % retained** | Rho: -0.594 p=0.004  FDR: p=0.013 | Rho: -0.397 p=0.075  FDR: p=0.143 | Rho: -0.585 p=0.005  FDR: p=0.009 | Rho: -0.611 p=0.003  FDR: p=0.006 | Rho: -0.333 p=0.140  FDR: p=0.242 |
| **BVMT-R immediate** | Rho: -0.332 p=0.142  FDR: p=0.225 | Rho: -0.194 p=0.400  FDR: p=0.507 | Rho: -0.539 p=0.012  FDR: p=0.018 | Rho: -0.620 p=0.003  FDR: p=0.006 | Rho: -0.211 p=0.359  FDR: p=0.457 |
| **BVMT-R delayed** | Rho: -0.356 p=0.113  FDR: p=0.195 | Rho: -0.234 p=0.308  FDR: p=0.418 | Rho: -0.602 p=0.004  FDR: p=0.008 | Rho: -0.659 p=0.001  FDR: p=0.006 | Rho: -0.315 p=0.165  FDR: p=0.261 |
| **BVMT-R discrimination index** | Rho: -0.229 p=0.319  FDR: p=0.433 | Rho: -0.264 p=0.248  FDR: p=0.362 | Rho: -0.546 p=0.01  FDR: p=0.016 | Rho: -0.559 p=0.008  FDR: p=0.014 | Rho: -0.368 p=0.10  FDR: p=0.190 |
| **BVMT-R % retained** | Rho: -0.205 p=0.374  FDR: p=0.444 | Rho: -0.138 p=0.551  FDR: p=0.616 | Rho: -0.447 p=0.042  FDR: p=0.047 | Rho: -0.514 p=0.017  FDR: p=0.025 | Rho: -0.509 p=0.018  FDR: p=0.190 |
| **KIMT** | Rho: -0.178 p=0.441  FDR: p=0.493 | Rho: -0.276 p=0.226  FDR: p=0.358 | Rho: -0.448 p=0.042  FDR: p=0.047 | Rho: -0.337 p=0.135  FDR: p=0.151 | Rho: -0.287 p=0.208  FDR: p=0.304 |
| **KAMT** | Rho: 0.041 p=0.861  FDR: p=0.909 | Rho: -0.053 p=0.819  FDR: p=0.819 | Rho: -0.284 p=0.212  FDR: p=0.364 | Rho: -0.283 p=0.214  FDR: p=0.214 | Rho: -0.200 p=0.385  FDR: p=0.457 |
| **Abbreviations:** BVMT-R: Brief Visuospatial Memory Test-Revised; HDGECs: Huntington’s Disease gene expansion carriers; HC: Healthy Controls; HVLT-R: Hopkins Verbal Learning Test – Revised; KAMT: Kinney’s Affect Matching Test; KIMT: Kinney’s Identity Matching Test; MoCA: Montreal Cognitive Assessment; TMT: Trail Making Test. Pearson’s partial correlation analysis controlling for age and sex. In red are the significant *p* values after FDR correction. | | | | | |
